# Controllable Preparation of rGO-PPS Composite Filter Material Based on Spray Modification and Its Filtration Performance and Dust-Cleaning Effect

**DOI:** 10.3390/ma19081670

**Published:** 2026-04-21

**Authors:** Xin Zhang, Ming Li, Huiying Tian, Daehyeon Kim, Yong Jin

**Affiliations:** 1 College of Architecture and Energy Engineering, Wenzhou University of Technology, Wenzhou 325000, China; zhangxin17@xauat.edu.cn; 2 Shandong Key Laboratory of Intelligent Manufacturing Technology for Advanced Power Equipment, Weifang University, Weifang 261061, China; tianwei666_2000@163.com; 3 Shenzhen SDG Service Co., Ltd., Shenzhen 518034, China; lililililiming@163.com; 4 School of Machinery and Automation, Weifang University, Weifang 261061, China; 5 Department of Civil Engineering, Chosun University, Gwangju 61452, Republic of Korea; dkimgeo@chosun.ac.kr

**Keywords:** spray modification, rGO-PPS composite filter material, filtration performance, dust-cleaning effect, experiment

## Abstract

With the continuous promotion of the dual carbon target, effective control of high-concentration dust pollutants in industrial sites is of great value for the healthy creation of healthy industrial environments and efficient energy utilization. In this study, we used the spraying method to improve and prepare the dust removal material, polyphenylene sulfide (PPS) fiber filter material, and test the filtration performance, resistance characteristics, and dust-cleaning effect of the improved rGO-PPS material. The results showed that, compared with PPS filter material, rGO-PPS material significantly improved particle filtration efficiency, with a filtration efficiency 0.058–19.417% higher in the particle size range of 0.265–5.75 μm. The higher the spraying concentration of the composite filter material, the higher the filtration efficiency at the same particle size. The comprehensive filtration performance of rGO-PPS composite filter material with a concentration of 3 g/L was better, as it better met the requirements of “high efficiency and low resistance”. With an increase in dust load, the filtration resistance of the filter material showed a continuous upward trend. The dust peeling rate increased with an increase in blowback wind speed. When the blowback wind speed reached 0.3 m/s, the dust-cleaning effect of the filter material tended to stabilize. Under this condition, the dust peeling rate of PPS filter material was 61.58%, and the dust peeling rate of 3 g/L rGO-PPS composite filter material reached 74.52%. These research results provide an experimental basis and technical support for the development and engineering application of high-efficiency purification filter materials for industrial multi-source pollutants.

## 1. Introduction

With China’s comprehensive promotion of the dual carbon strategy of “carbon peak and carbon neutrality”, the industrial sector, as the core sector of energy consumption and carbon emissions, has become key to achieving these dual carbon goals due to its green and low-carbon transformation [1]. In the industrial production process, industries such as metallurgy, chemical engineering, building materials, and electricity generate a large amount of dust pollutants [2,3,4]. Among them, high concentrations of particulate matter not only cause atmospheric pollution and harm the ecological environment and human health, but also affect the stable operation of industrial production equipment and increase equipment maintenance costs and energy consumption [5,6]. Among them, particulate matter is divided into PM10 (particles ≤ 10 μm), PM2.5 (particles ≤ 2.5 μm), and PM1.0 (particles ≤ 1.0 μm) [3]. PM10 is inhalable particulate matter, which can enter the respiratory tract; PM2.5 is fine particulate matter, which can penetrate into the bronchi and alveoli; PM1.0 is ultrafine particulate matter, which can enter the blood circulation and cause the greatest harm to human health. In industrial flue gas, PM1.0 and PM2.5 are the key control objects due to their strong diffusivity, strong adhesion, and difficulty of removal. Bag filter technology, with its advantages of high dust removal efficiency, stable operation, and wide applicability, has become a mainstream technology for the purification of industrial pollutants [7]. As the core component of bag filter systems, the performance of filter material directly determines the purification effect, energy consumption level, and service life of the entire dust removal system [8].

Polyphenylene sulfide (PPS) fiber has excellent heat resistance, chemical corrosion resistance, and mechanical stability, and it can adapt to harsh industrial flue gas environments such as high temperatures and acid–base corrosion. It is a commonly used filter material substrate in the field of high-temperature flue gas filtration [9]. However, PPS fibers have low surface energy and insufficient surface roughness, which limits their ability to intercept and adsorb fine particles. During long-term operation, problems such as decreased filtration efficiency, a rapid increase in operating resistance, and incomplete dust cleaning often occur [10,11], resulting in increased energy consumption of the dust removal system and a certain difference from the development needs of energy conservation, consumption reduction, and efficient purification under the dual carbon target. Therefore, optimizing the surface structure and interface characteristics of PPS filter material through reasonable modification methods, while ensuring low resistance, to improve filtration efficiency and ash removal performance, has important engineering value and practical significance for promoting the upgrading of industrial flue gas purification technology and assisting in carbon reduction in the industrial field.

In recent years, scholars, both domestically and internationally, have conducted extensive research on the modification of high-temperature filter material [12,13,14,15]. The modification methods mainly focused on surface coating [12], electrospinning [13], plasma treatment [14], and nanomaterial loading [15]. Among them, nanocarbon materials are widely used for the functional modification of filter material due to their large specific surface area, abundant surface active sites, and excellent chemical stability [16]. Reduced graphene oxide (rGO) retains the high specific surface area and rich functional groups of graphene oxide while repairing some conjugated structures. Its conductivity and mechanical properties are improved, and it has shown great potential for applications in separation fields such as air filtration and water treatment [17,18].

In existing research, scholars have introduced rGO into filter material systems through immersion coating, in situ synthesis, electrospinning, and other methods, confirming that rGO can effectively improve the surface microstructure of filter material and enhance its ability to intercept fine particles [19]. However, for some modification processes, problems such as complex processes, high reagent consumption, weak bonding strength of the modified layer, and easy detachment are encountered, making the achievement of industrial mass production difficult [20]. At the same time, existing research mostly focuses on the single performance analysis of filtration efficiency and resistance, and systematic research on the dust deposition law, resistance evolution characteristics, and back-blowing cleaning behavior of modified filter material in actual operation is relatively scarce [21]. In addition, there are still certain shortcomings in the optimization of rGO spray modification process parameters for PPS fibers, especially in terms of the correlation between spray concentration, particle size, and comprehensive performance of filter material, as well as the mechanism of the influence of back-blowing wind speed on the dust stripping rate during the cleaning process.

In response to the shortcomings of existing research, this study adopts a green and efficient spraying process to modify the surface of PPS fiber filter material with rGO and prepare rGO-PPS composite filter material. The influence of different particle sizes and spraying concentrations on the microstructure of composite filter materials is explored via a comparison of the filtration performance and dust-cleaning effect of the testing system under different spraying concentrations. The filtration efficiency and operating resistance of PPS filter material and rGO/PPS composite filter material, as well as the correlation between dust load and filter material resistance, the influence of backwash air velocity on the dust peeling rate of filter material, and the quantitative analysis of the influence of backwash air velocity on the dust peeling rate, provide data support for the practical application of modified filter material in industrial bag dust removal systems.

## 2. Methods

### 2.1. Experimental Materials

**Figure 1:**
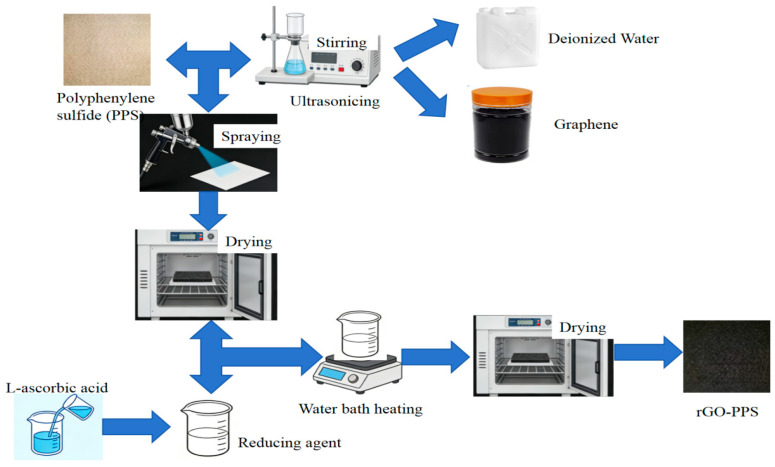
Preparation process.

We used polyphenylene sulfide (PPS) fiber filter material as the matrix. This was provided by Botou Tianqing Environmental Protection Machinery Equipment Co., Ltd. (Botou, China). The commercial polyphenylene sulfide (PPS) fiber filter material used in this study was linear and of high-thermal-stability grade, designed for high-temperature flue gas filtration. The average molecular weight (Mw) of the PPS resin was approximately 45,000–55,000 g/mol, with a polydispersity index (PDI) of 1.3–1.5. Chemical structure: PPS fiber has a linear macromolecular structure with a repeating unit of –(C_6_H_4_–S)–, without obvious branching or excessive crosslinking. Graphene was provided by Suzhou Carbon Feng Graphene Technology Co., Ltd. (Suzhou, China); deionized water was provided by Hangzhou Yongjieda Purification Technology Co., Ltd. (Hangzhou, China); and L-ascorbic acid was provided by Tianjin Kemio Chemical Reagent Co., Ltd. (Tianjin, China). We used a spray-coating method to form a layer of reduced graphene oxide coating on the dust-facing surface of the polyphenylene sulfide fiber filter material. The specific steps are as follows, and the preparation flowchart is shown in Figure 1:

(1) Cut the polyphenylene sulfide fiber into 7 cm × 7 cm samples → weigh and record.
(2) Prepare 1 g/L, 3 g/L, and 5 g/L graphene/deionized water solutions.
(3) Ultrasonicate at 500 W for 60 min at room temperature.
(4) Spray onto dust-facing surface (5 cm distance, vertical, constant moving speed).
(5) Dry at 80 °C for 120 min to obtain graphene oxide composite filter material.
(6) Immerse in 0.2 mol/L of L-ascorbic acid solution (bath ratio 50:1) at 70 °C for 60 min.
(7) Dry at 80 °C for 120 min.
(8) Conduct tape peeling + weight gain uniformity + SEM to verify adhesion and uniformity.

**Figure 2:**
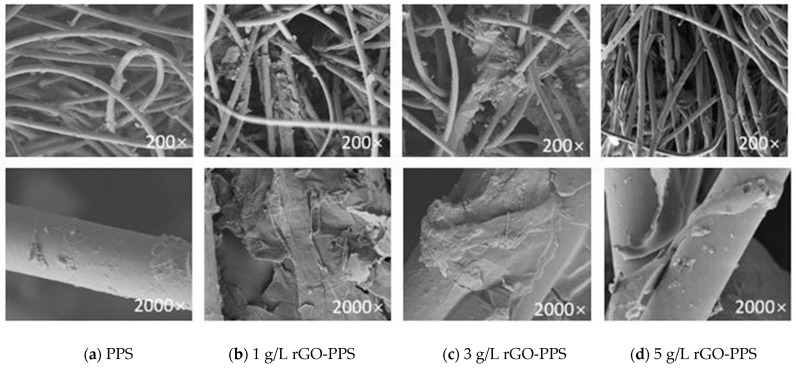
SEM images of different materials [22].

The PPS fiber used was linear, non-crosslinked, with Mw ≈ 45,000–55,000 g/mol. Its structure enabled strong π–π interactions with rGO, supporting stable coating formation and good composite performance. These characteristics did not negatively affect filtration efficiency or dust-cleaning behavior. Scanning electron microscopy (SEM) was used to characterize the surface morphology of PPS and rGO-PPS composite filter materials. The PPS fiber surface was smooth and flat; after 1 g/L rGO spraying, a small amount of rGO nanosheets was attached to the fiber surface; after 3 g/L rGO spraying, a uniform and dense rGO coating was formed on the fiber surface, with obvious wrinkles and protrusions; after 5 g/L rGO spraying, excessive rGO nanosheets accumulated locally on the fiber surface, and partial pore blockage occurred. The above results are consistent with our previous work (Figure 2) [22]. The scanning electron microscopy (SEM) data were obtained from the preliminary work of Li Ming [22], one of the authors of this paper, and are cited in this paper.

### 2.2. Experimental Apparatus

**Figure 3:**
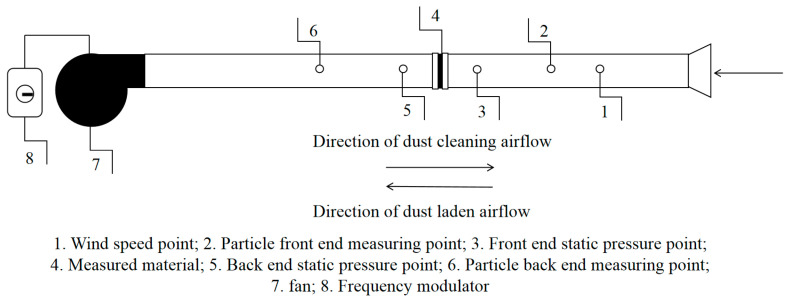
Experimental setup.

The GRIMM1.109 aerosol spectrometer was used to measure the mass concentration and quantity concentration of particulate matter at the front and rear ends of the filter material. The sampling flow rate was 1.2 L/min, and the measurement range of particulate matter mass concentration was 0.1–100,000 μg/m^3^. The particle counting concentration range reached 2,000,000 P/L, with a repetition rate of 5%. The HD37AB1347 multiparameter air-quality monitor was used to measure temperature, humidity, and wind speed in the experimental environment, supplied by DeltaOHM Co., Ltd., Selvazzano (PD), Italy. The wind speed measurement range was 0–50 m/s, the temperature measurement range was −70–+400 °C, and the humidity measurement range was 0–100% RH, with resolutions of wind speed of 0.01 m/s, temperature of 0.1 °C, and humidity of 0.1% RH. The HD2114P.0 digital micro pressure gauge was used to measure the resistance at both ends of the material, supplied by DeltaOHM Co., Ltd., Selvazzano (PD), Italy, with a pressure measurement range of 0–2000 Pa, a pressure resolution of 0.5 Pa, and a differential pressure measurement accuracy of 0.4% FS of the full range. The XCS-101-0BS electric blast drying oven was used for drying, and it was supplied by Shaoxing shi Shang Cheng Instrument equipment Co., Ltd., Shaoxing, China, with a temperature adjustment range from room temperature to 300 °C. An ultrasonic cleaning machine (KQ-500DE, Kunshan Ultrasonic Instrument, Kunsha, China), with an adjustable power range of 0–500 W, was used. The constant-temperature water bath was provided by Shanghai Lichen Bangxi Instrument Technology Co., Ltd. (Shanghai, China), model HH-2. Temperature setting range: room temperature +5–100 °C; temperature resolution: 0.1 °C. Each sample was tested in 3 groups, with a total of 12 groups consisting of 4 samples. The average concentration value before and after the test filter time of 5 min was used as the calculated value for each group to reduce experimental errors. The experimental platform is shown in Figure 3. Based on the results of previous research and the use of a comparative experimental design, this study comprehensively considered [22,23,24] and selected talc powder as the experimental dust. Talc powder has the characteristics of strong controllability of particle dispersion, high stability of dust source concentration, and uniform physical and chemical properties [25], which help to reduce the interference of experimental variables and improve the reproducibility and reliability of the test results, proving that using it as experimental dust is more representative and meets experimental needs [24]. This is achieved through turning on the fan and dust-generating device, adjusting the frequency converter to determine the dust loading time, and systematically studying its filtration effect. For the dust-cleaning experiment, the frequency converter is adjusted to control the back-blowing wind speed, the back-blowing airflow is set in the pipeline, and the back-blowing time is adjusted through the fan switch. The back-blowing duration is 15 s, and the system studies its impact on the dust-cleaning effect.

### 2.3. Performance Parameters

The filtration resistance is calculated according to Formula (1) [26]:

(1) $$\Delta P=P_{2}-P_{1}$$

where ΔP is the filtration resistance, Pa; *P*_1_ is the static pressure of the front-end pipeline of the filter material, Pa; and *P*_2_ is the static pressure of the back-end pipeline of the filter material, Pa.

The filtration efficiency is calculated according to Formula (2) [26]:

(2) $$\eta \left(\%\right)=\frac{C_{1}-C_{2}}{C_{1}}\times 100$$

where η is the filtration efficiency, *C*_1_ is the concentration of particulate matter before filtration (μg/m^3^), and *C*_2_ is the concentration of particulate matter after filtration (μg/m^3^).

The dust peeling rate is calculated according to Formula (3) [22]:

(3) $$\eta \left(\%\right)=\frac{m_{1}-m_{2}}{m_{1}}\times 100$$

where *m*_1_ is the dust load before dust cleaning, kg/m^2^; *m*_2_ is the dust load after dust cleaning, kg/m^2^.

The dust peeling rate is also calculated according to Formula (4) [22]:

(4) $$\eta =\frac{P_{1i}-P_{2i}}{P_{1i}}\times 100\%$$

where *P*_1*i*_ is the resistance of the filter bag before dust cleaning, Pa; *P*_2*i*_ is the resistance of the filter bag after dust cleaning, Pa. In this study, the weighing method was used to determine the relationship between the blowback wind speed and the dust peeling rate of PPS filter material and rGO composite filter material when the dust load was 1 kg/m^2^.

## 3. Results and Discussion

### 3.1. Resistance Variation in Filter Material

**Figure 4:**
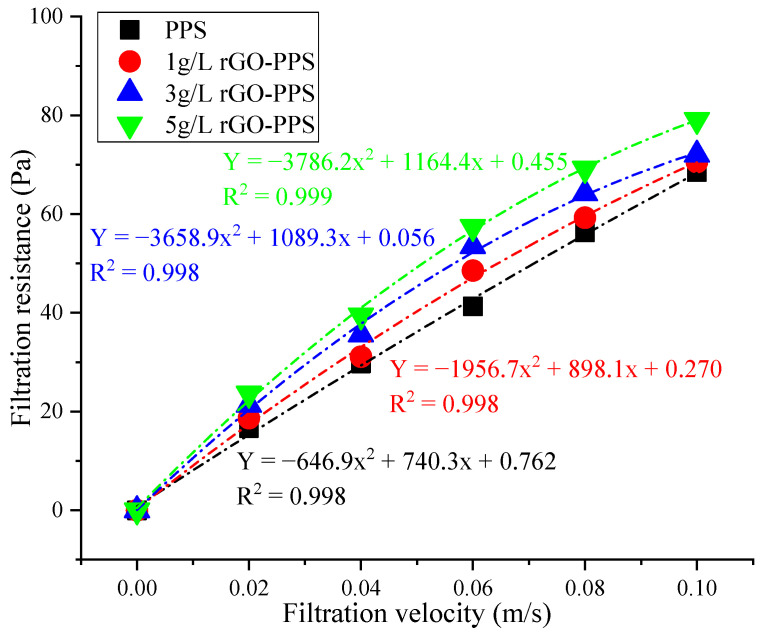
Changes in filtration resistance.

The changes in filtration resistance and filtration velocity of different filter material are shown in Figure 4.

From Figure 4, it can be seen that, within the range of filtration velocity from 0 to 0.10 m/s, the filtration resistance of different filter materials shows a significant upward trend with the increase in filtration velocity. The filtration resistance range of PPS filter material is 16.64–68.51 Pa, the filtration resistance range of 1 g/L rGO-PPS composite filter material is 18.65–70.53 Pa, the filtration resistance range of 3 g/L rGO-PPS composite filter material is 21.34–72.11 Pa, and the filtration resistance range of 5 g/L rGO-PPS composite filter material is 23.62–79.08 Pa, and all datasets conform to a quadratic function fitting relationship (R^2^ ≥ 0.998), indicating a significant nonlinear positive correlation between resistance and velocity [27]. At the same filtration velocity, the filtration resistance of the composite filter material increases with the increase in the rGO loading concentration, showing a trend of 5 g/L rGO-PPS > 3 g/L rGO-PPS > 1 g/L rGO-PPS > PPS. The main reason for this is that rGO nanosheets are constructed of a denser three-dimensional network structure on the surface of the PPS fibers [28], making the modified fiber surface rough with more protrusions and wrinkles, increasing the tortuosity and effective specific surface area of the airflow passing through the filter material [28], thereby strengthening the friction and impact between the airflow and fibers, which is consistent with the results in the literature [29], contributing to the verification of this study. The fiber surface of PPS filter material is relatively smooth, and the airflow can pass through smoothly, so the resistance is relatively low. With the increase in filtration velocity, the Reynolds number of the airflow increases, the turbulence effect is enhanced, and the rough surface of the rGO-modified layer induces more vortices and boundary layer separation [30], further exacerbating energy consumption, resulting in a significant increase in the rate of resistance with increasing velocity, which is consistent with the quadratic coefficient in the fitted curve increasing with an increasing rGO concentration. In addition, as the rGO loading concentration increases from 1 g/L to 5 g/L, the porosity inside the filter material decreases, the pore size distribution becomes narrower, and the resistance to airflow increases accordingly. Therefore, although the introduction of rGO inevitably increases filtration resistance, its high specific surface area and strong adsorption characteristics can significantly enhance the capture efficiency of filter material for submicron particles [31]. The resistance curves of 3 g/L and 5 g/L rGO-PPS are highly similar, and a balance between filtration performance and energy consumption cost needs to be found in the actual process.

### 3.2. The Influence of Different Particle Sizes on Filtration Efficiency

**Figure 5:**
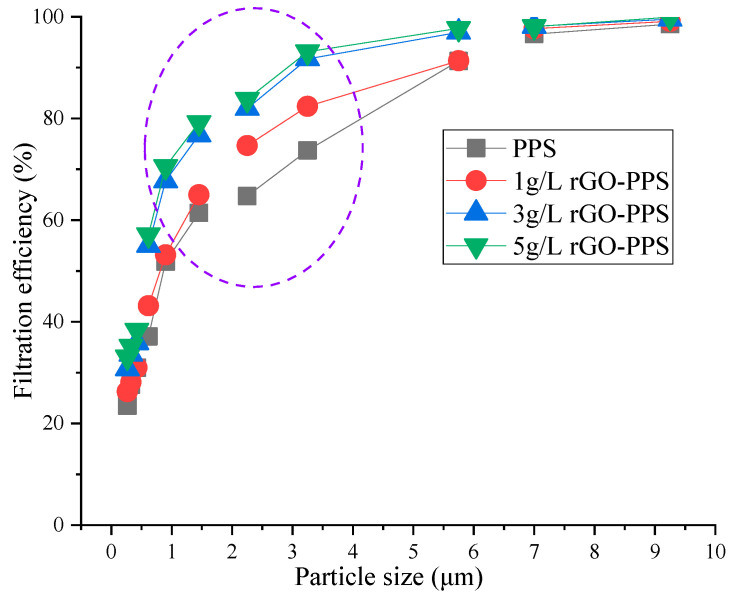
Filtration efficiency of different particle sizes.

The counting efficiency changes in different particle sizes under different filter materials at a filtration velocity of 0.1 m/s were tested using atmospheric aerosols as the dust source, as shown in Figure 5.

As demonstrated in Figure 5, it can be seen that, under different concentrations of rGO spray, the filtration efficiency of the composite filter material significantly increases with an increase in particle size, and the filtration performance of the rGO-modified filter material (1 g/L, 3 g/L, 5 g/L rGO-PPS) is better than that of the unmodified PPS filter material. For particles with a particle size of less than 2.0 μm, composite filter material with different rGO concentrations showed significant performance advantages. Among them, 5 g/L rGO-PPS had the highest filtration efficiency, followed by 3 g/L rGO-PPS, 1 g/L rGO-PPS, and PPS filter material, which had the lowest efficiency. This indicates that the introduction of rGO effectively enhances the capture ability of the filter material for fine particles, and the higher the spraying concentration, the more significant the improvement effect. As the particle size increases to over 5.0 μm, the filtration efficiency of all filter material approaches 100%, and the differences between different materials gradually decrease. This is because large particles are more easily captured by the filter material through mechanisms such as inertial collision and interception, and the influence of the modification of the filter material itself on the filtration efficiency of large particles is weakened [32].

**Figure 6:**
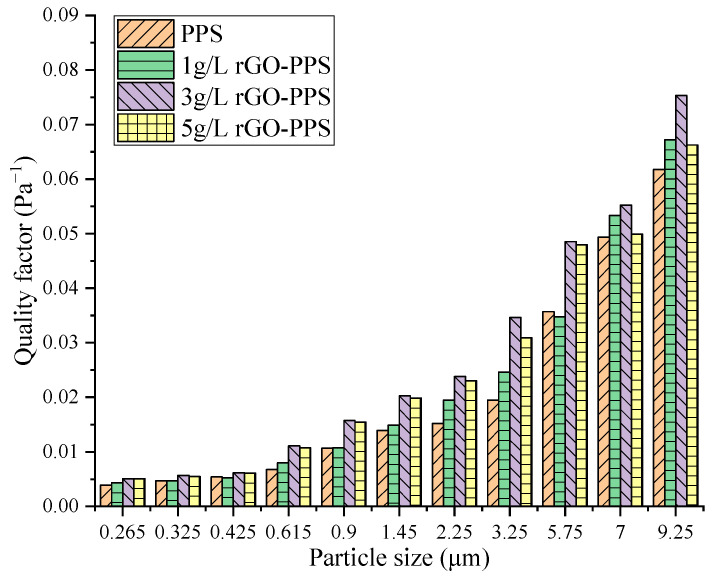
Differences in quality factors.

Overall, rGO modification significantly improved the filtration performance of PPS filter material for fine particulate matter, with a filtration efficiency 0.374–19.417% higher in the particle size range of 0.265–5.75 μm. The particle size range of 0.265–5.75 μm covers PM1.0, PM2.5, and part of PM10. The higher the spraying concentration of the composite filter material, the higher the filtration efficiency at the same particle size. At a particle size of 0.9 μm, the difference was the largest, with a difference of 17.366% between 5 g/L rGO-PPS and 1 g/L rGO-PPS, 2.784% between 5 g/L rGO-PPS and 3 g/L rGO-PPS, and 14.582% between 3 g/L rGO-PPS and 1 g/L rGO-PPS. To further determine the relatively optimal spray concentration and achieve a balance between filtration efficiency and resistance characteristics, the quality factors of different filter materials are shown in Figure 6.

Figure 6 shows the variation in the quality factors of four types of filter materials with particle sizes in the range of 0.265–9.25 μm. Overall, the quality factors of all filter materials show a continuous upward trend with the increase in particle size, and the performance differences between rGO-PPS composite filter materials with different modified concentrations and PPS filter material gradually become prominent with the change in particle size. Raw PPS filter material’s quality factor remains at the lowest level throughout the entire particle size range, especially in the fine particle size range (<3.25 μm), with the most significant difference compared with rGO-modified filter material. The quality factor of 1 g/L rGO-PPS filter material has been improved compared with PPS filter material, but it is significantly lower than 3 g/L and 5 g/L rGO-PPS in the entire particle size range. The 5 g/L rGO-PPS filter material only slightly dominates at the maximum particle size (9.25 μm), and its quality factor is lower than 3 g/L rGO-PPS in the fine particle range below 5.75 μm. The 3 g/L rGO-PPS filter material exhibits the highest quality factor in the vast majority of particle size ranges, especially in the 3.25 μm and above particle size range that is of particular concern in engineering applications, demonstrating significant comprehensive performance advantages.

The main reason for these results is that the nanolayer structure of rGO provides abundant physical interception and adsorption sites for particle capture [17,18], which is the core reason for the improvement in the efficiency of modified filter material. This increased the filtration efficiency of rGO-PPS composite filter material for fine particles ranging from 0.265 to 5.75 μm by 0.374% to 19.417% compared with PPS filter material. But when the concentration of rGO spray reaches 5 g/L, excess rGO will form a stack on the surface of the filter material, blocking some of the microporous channels [28], resulting in a limited increase in its capture efficiency for fine particles, and instead making the concentration lower than 3 g/L rGO-PPS. In addition, the filtration resistance of the filter material increases with the increase in the rGO spraying concentration, and the core reason for this is that the increase in rGO loading will reduce the surface porosity of the filter material [31]. Among them, the efficiency improvement of 1 g/L in the modified concentration is limited, while a high concentration of 5 g/L causes a sharp increase in resistance, greatly offsetting its efficiency advantage. Only 3 g/L rGO-PPS can achieve a significant efficiency improvement while controlling the increase in resistance within a reasonable range.

The quality factor, as a core indicator for the comprehensive evaluation of filtration efficiency and resistance characteristics, directly reflects the degree of adaptation of the filter material to high efficiency and low resistance. In summary, the comprehensive filtration performance of rGO composite filter material with a concentration of 3 g/L is better and more in line with the requirements of “high efficiency and low resistance”.

### 3.3. The Influence of Dust Load and Filtration Resistance

**Figure 7:**
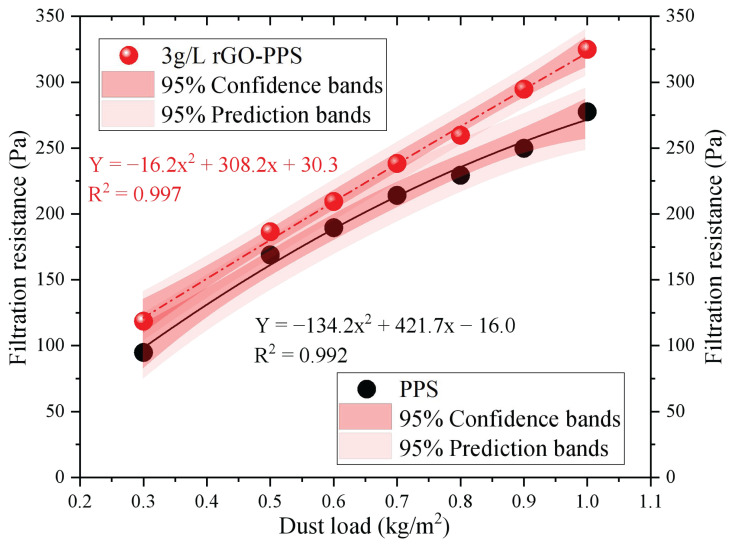
Changes in filtration resistance and dust load.

Under the experimental wind speed of 1.0 m/s and 3 g/L rGO-PPS, the operating resistance of PPS filter material and 3 g/L rGO-PPS composite filter material was tested under different dust loads. The results are shown in Figure 7.

From Figure 7, it can clearly be seen that, with an increase in dust load, the filtration resistance of both filter materials shows a continuous upward trend. By fitting the experimental data with a quadratic polynomial, the R^2^ of 3 g/L rGO-PPS was found to be 0.997, while the R^2^ of PPS was 0.992. The high fitting degree indicates that the model can accurately reflect the variation in resistance with dust load for both. When the dust load is 0.3 kg/m^2^, the filtration resistance of 3 g/L rGO-PPS is 118.62 Pa, while that of PPS filter material is 94.87 Pa, with a small difference of 23.75 Pa. When the dust load is increased to 1.0 kg/m^2^, the filtration resistance of 3 g/L rGO-PPS is about 324.95 Pa, while that of PPS filter material reaches about 277.56 Pa, and the difference in resistance expands to 47.39 Pa, an increase of about 49.88%. This indicates that with the increase in dust load, the resistance growth rate of PPS filter material is significantly faster than that of 3 g/L rGO-PPS composite filter material. The main reason for this is that, after rGO modification, the physical and chemical properties of the filter material surface change. The hydrophobic properties and smooth structure of rGO nanosheets effectively suppress the adhesion and aggregation of dust particles on the filter material surface [33], making the formed dust layer more porous and loose, thereby significantly reducing the additional resistance of the dust layer. In addition, the high specific surface area and electrostatic adsorption of rGO make the distribution of dust particles on the surface of the filter material more uniform [34], reducing the occurrence of local blockage. The surface polarity of PPS raw filter material is strong, and dust particles are prone to agglomeration and accumulation, forming a dense dust layer, resulting in a sharp increase in filtration resistance with increasing dust load. Therefore, the 3 g/L rGO-PPS composite filter material can effectively delay the growth rate of filtration resistance, reduce the frequency of ash cleaning and operating energy consumption during long-term operation, and demonstrate better dynamic filtration performance and industrial application potential. The rGO loading of the composite filter material was quantified by the weighing method. The rGO loadings of 1 g/L, 3 g/L, and 5 g/L spray concentrations were 0.21 wt%, 0.63 wt%, and 1.05 wt%, respectively. The 3 g/L concentration has the most uniform coating and no obvious agglomeration, which is consistent with the optimal comprehensive performance.

### 3.4. The Relationship Between Blowback Wind Speed and Dust-Cleaning Effect

**Figure 8:**
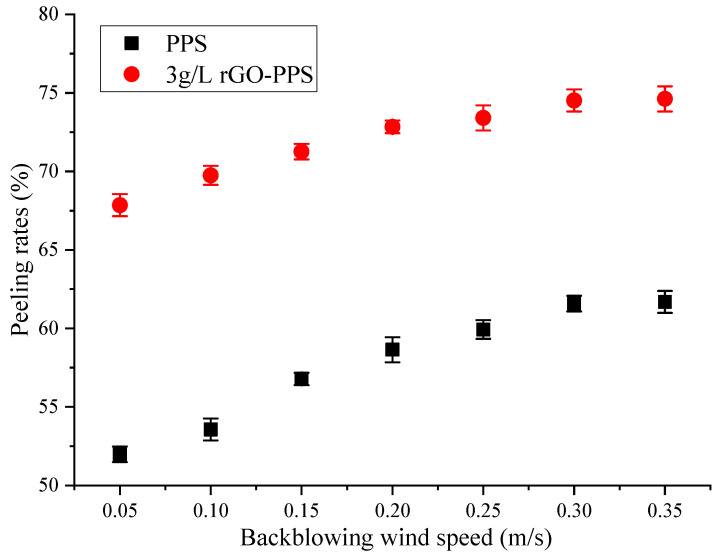
Changes in back-blowing wind speed and dust-cleaning efficiency.

Under the condition of a dust load of about 1 kg/m^2^, the dust peeling rate of PPS filter material and 3 g/L rGO-PPS composite filter material was tested at different blowing speeds, and the results are shown in Figure 8.

From Figure 8, it can clearly be seen that the dust peeling rate of both filter materials increases with the increase in blowback wind speed, and grows rapidly in the range of 0.05–0.30 m/s. When the blowback wind speed reaches 0.30 m/s, the peeling rate growth tends to be flat. When the back-blowing wind speed is 0.05 m/s, the dust peeling rate of PPS filter material is about 51.98%, while the 3 g/L rGO-PPS composite filter material can reach 67.85%, with a difference of 15.87% between the two. When the back-blowing wind speed is increased to 0.30 m/s, the dust peeling rate of PPS filter material is 61.58%, while the 3 g/L rGO-PPS composite filter material can reach 74.52%, with a difference of 12.94%. This indicates that the cleaning effect of the 3 g/L rGO-PPS composite filter material is significantly better than that of the PPS original filter material in the entire wind speed range, and has greater potential for improving cleaning efficiency. The main reason for this is that, after rGO modification, the physical and chemical properties of the filter material surface change, and a layer of reduced graphene oxide coating is deposited in the fibers, similar to the mirror treatment [35]. The hydrophobic properties and low surface energy of rGO nanosheets effectively reduce the adhesion between dust particles and filter material fibers [33], making it easier for the dust layer to peel off from the filter material surface under the action of reverse airflow. The surface of PPS filter material is filled with attached fluff [36], increasing the contact area, while the surface of dust particles generally carries polar groups or charges [37], making it easy for dust particles to adhere and aggregate. Moreover, PPS filter material has a higher porosity, making it easier for dust to enter the interior of the filter material, resulting in increased difficulty in peeling off. In addition, the high specific surface area and electrostatic adsorption of rGO make the distribution of dust particles on the surface of the filter material more uniform [34], resulting in a looser and more porous dust layer. The back-blowing airflow is more likely to penetrate the dust layer and generate sufficient stripping force. The surface of PPS filter material is prone to forming a dense dust layer, resulting in high resistance to airflow penetration and lower dust peeling rate. Therefore, the 3 g/L rGO-PPS composite filter material can achieve a higher dust peeling rate with lower back-blowing wind speed during the dust-cleaning process, demonstrating excellent dust-cleaning performance and wider applicability in practical applications.

### 3.5. Comparison with Existing Filtration Materials

This gives rGO-PPS a wider service range than non-heat-resistant filter materials (e.g., PET) and better functionality than traditional PPS. Table 1 presents the comparison results of differences between other materials and composite materials under different parameters.

As clearly shown in Table 1, the 3 g/L rGO-PPS composite prepared via spray modification in this study outperforms all reported modified PPS/PET filter materials in fine-particle filtration efficiency improvement, dust peeling rate, and quality factor balance. Unlike plasma-treated PPS with limited efficiency enhancement (<10%) and nanomaterial-coated PPS with sharp resistance rise, the 3 g/L rGO-PPS achieved a maximum 19.417% efficiency improvement for 0.265–5.75 μm particles while controlling resistance growth within a reasonable range. Additionally, the 74.52% dust peeling rate was the only quantified high-value result among reported PPS-based modified materials, and the spray modification process avoids the complex steps, high cost, and poor coating fastness of electrospinning, in situ synthesis, and dip-coating methods [41,42]. Combined with the inherent heat resistance and chemical stability of PPS, the rGO-PPS composite fills the gap between high-performance filtration and practical engineering application for high-temperature flue gas filter materials, and its scalability further supports industrial mass production. In addition, with continuous underground mining, the differences in underground environments will also increase the demand for safe working environments [43,44,45,46]. Effective ventilation and dust removal for complex underground environments will be one of the necessary factors to ensure the health and safety of working environments in the future.

## 4. Conclusions

In this study, we use spray modification technology to prepare a rGO-PPS composite filter material with different concentrations, and we systematically explore its filtration performance, resistance characteristics, and dust-cleaning effect. Through comparative experiments with pure PPS filter material, the core conclusions of this study are as follows:

1. Within the range of 0–0.10 m/s filtration velocity, the filtration resistance of all filter materials significantly increased with an increase in velocity and conformed to the quadratic function fitting relationship (R2 ≥ 0.998). At the same filtration velocity, the resistance of the composite filter material increased with an increase in the rGO spraying concentration (5 g/L rGO-PPS > 3 g/L rGO-PPS > 1 g/L rGO-PPS > PPS), because the dense three-dimensional network structure constructed by rGO increases the tortuosity of the airflow channel and reduces the porosity of the filter material.
2. Within the particle size range of 0.265–5.75 μm, the filtration efficiency of rGO-PPS composite filter material was 0.058–19.417% higher than that of PPS filter material, and the higher the spraying concentration, the higher the filtration efficiency under the same particle size. When the particle size was greater than 5.0 μm, the filtration efficiency of all filter materials approached 100%, and the difference caused by modification gradually decreased. The 3 g/L rGO-PPS composite filter material showed the best comprehensive performance, achieving a significant improvement in fine particle filtration efficiency while controlling the increase in resistance within a reasonable range, which meets the core requirements of “high efficiency and low resistance” in dust removal.
3. As the dust load increased, the filtration resistance of both PPS filter material and 3 g/L rGO-PPS composite filter material continued to rise, but the growth rate of the PPS filter material resistance was significantly faster. The hydrophobic properties, low surface energy, and electrostatic adsorption of rGO make the dust layer more porous and evenly distributed, effectively suppressing dust adhesion, aggregation, and local blockage and reducing the additional resistance of the dust layer.
4. The dust peeling rate of both filter materials increased with the increase in blowback wind speed. The peeling rate increased rapidly in the range of 0.05–0.30 m/s, and the cleaning effect tended to stabilize after the blowback wind speed reached 0.30 m/s. Under this working condition, the dust peeling rate of PPS filter material was 61.58%, and the 3 g/L rGO-PPS composite filter material reached 74.52%. The rGO coating reduces the adhesion between dust and filter material, and the loose dust layer formed is more easily peeled off by the reverse airflow, resulting in better dust-cleaning performance in the entire wind speed range.

## 5. Limitations and Future Research Directions

Although this study provides important experimental evidence for the industrial application of rGO-PPS composite filter material, some limitations still exist, such as the single experimental conditions, non-orthogonal optimization of modified process parameters, lack of long-term operational performance testing, insufficient analysis of micromechanisms, and failure to consider industrial economics. Subsequent research can simulate the actual complex flue gas environment in industries such as metallurgy and chemical engineering to conduct performance testing; optimize entire process parameters such as spray concentration, pressure, and drying temperature through orthogonal experiments; and conduct multiple aging experiments of filtration cleaning cycles to explore the long-term operational stability of filter material. Through the use of characterization methods such as SEM and XPS, combined with molecular dynamics simulations, rGO and PPS can be analyzed in depth. In the assessment of the micromechanism interaction between fibers and dust particles, as well as conducting large-scale pilot experiments on filter material and completing full lifecycle economic analysis, photocatalytic nanomaterials can also be introduced to develop functional composite filter material for the synergistic purification of multiple pollutants, the recycling and regeneration process of waste filter material can be studied, a closed-loop industrial chain can be formed, and the standardized preparation and industrial application of rGO composite filter material can be further promoted.

## Figures and Tables

**Table 1:** Comparative differences in pollen season with concurrent influenza circulation.

Material/ Composite	Modification/ Synthesis Mode	Concentration/Mass Loading	Target Particle Size Range (μm)	Filtration Efficiency Improvement	Dust Peeling Rate (%)	Quality Factor Performance
PPS	Unmodified	-	0.265–5.75	-	61.58 (0.3 m/s backwash)	Lowest across all particle sizes
3 g/L rGO-PPS	Spray modification + L-ascorbic acid reduction	3 g/L rGO spray concentration	0.265–5.75	0.058–19.417% vs. PPS	74.52 (0.3 m/s backwash)	Highest in 0.265–9.25 μm (especially <5.75 μm); optimal “high efficiency–low resistance” balance
Plasma-treated PPS [38,39]	Plasma surface treatment	-	Submicron–fine particles	<10% vs. PPS	Not reported	Moderate; efficiency improvement offset by minor resistance rise
TiO_2_/SiO_2_ nanomaterial-coated PPS [40]	Nanomaterial coating	-	Fine particles	~10–15% vs. PPS	Not reported	Low; sharp resistance increase (severe pore blockage)
Electrospun nanofiber-coated PPS [40]	Electrospinning nanofiber coating	-	Submicron particles	~12–18% vs. PPS	Not reported	Low; high resistance and poor coating fastness
PPS coated with polybenzoxazine [10]	Solution coating + curing	-	Industrial flue gas particles	~8–10% vs. PPS	Not reported	Moderate; improved hydrophobicity but limited efficiency
rGO-impregnated polyester (PET) [19]	Immersion impregnation	Unoptimized rGO loading	0.3–5.0	~5–12% vs. PET	Not reported	Moderate; high resistance for high rGO loading
Polydopamine-coated PET coarse filter [14]	Polydopamine surface coating	-	Fine particles	~6–9% vs. PET	Not reported	Low resistance but low efficiency

## Data Availability

The original contributions presented in this study are included in the article. Further inquiries can be directed to the corresponding author.
